# Conspicuous Chameleons

**DOI:** 10.1371/journal.pbio.0060021

**Published:** 2008-01-29

**Authors:** Kira E O'Day

Flashiness isn't an attribute we're used to associating with chameleons, but according to a new study of the evolution of chameleon color change by Devi Stuart-Fox and Adnan Moussalli, it's time to rethink the cliché of chameleons as unobtrusive critters who change color to blend with their surroundings.

While environmental cues can trigger temporary color changes in creatures as diverse as crabs, octopuses, and fish, chameleons are the most familiar color changers. Among the more than 150 chameleon species, the ability to change color ranges from subtle shifts in shades of brown to remarkable ensembles of orange, blue, green, and even ultraviolet colors that are invisible to humans. Investigations of the function of chameleon color change have yielded two main hypotheses. According to the camouflage (or crypsis) hypothesis, natural selection led to an increased ability of the chameleon to match a variety of backgrounds to escape predators. The alternative hypothesis proposes that color change evolved to facilitate social communication among chameleons of the same species. Under this scenario, color change enables chameleons to flash conspicuous color patterns that are highly detectable to other chameleons, while appearing camouflaged at other times.

To determine which selective pressures may have driven the evolution of color change, and toward which colors selection favored, Stuart-Fox and Moussalli compared the coloration and color-change capacities of 21 lineages of southern African dwarf chameleons (Bradypodion spp.) in a series of field-based behavioral trials. The authors chose to work with dwarf chameleons because the different species vary tremendously in the range and types of colors they can display, and because the evolutionary relationships within this group are known. Male dwarf chameleons use color to signal dominance to other males in aggressive contests and to woo females. If a male loses a contest or is vigorously rejected by a female, he displays an alternate set of submissive color patterns. Although chameleons also change color when confronted by a predator, because the color range is greatest when male dominant and submissive coloration is contrasted, the authors used this measure to estimate each species' capacity for color change.

To measure the degree to which increased capacity for color change correlates with crypsis or signal conspicuousness, Stuart-Fox and Moussalli first staged contests between male chameleons on a perch in their natural habitat and then took color measurements of the dueling chameleons and the surrounding vegetation using a technique called reflectance spectrometry, which measures the proportions of light wavelengths (and thus color) that reflect from surfaces. As soon as the chameleons showed either clear aggressive behavior—including head shaking, displays in which chameleons puff out their throats and flatten themselves to appear larger, or chasing and even biting —or submissive behaviors, such as fleeing or flipping to the underside of the branch, the researchers measured the color and brightness of the top, middle, and bottom portions of their flanks, along with other high-contrast regions (see the Figure). By pitting each male against several others, they obtained measurements for about five individuals in each of the 21 lineages, enabling them to measure species-specific color change. To measure background coloration, the authors took reflectance readings of the leaves, branches, grass, or vines where the chameleons were caught. They also took median reflectance measurements of the vegetation most common to each species. Using these data, along with data collected in previous studies regarding the spectral sensitivities of photoreceptors in the eyes of both chameleons and birds, the authors were able to estimate just how conspicuous each individual would be in a variety of environments, when viewed by both other chameleons and their bird predators.

**Figure:**
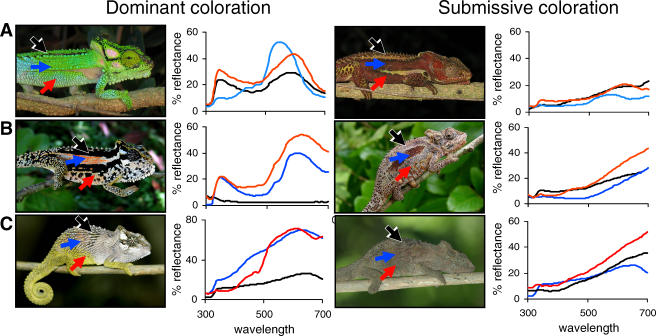
Displays and reflectance spectra for three species spanning the geographic range of the genus are shown.

Chameleon species that showed the greatest capacity for color change also had dominance signals that were most conspicuous to other chameleons—because they showed a highly contrasting color pattern and each color contrasts markedly with surrounding vegetation. There was no correlation (based on evolutionary relationships) between increased color change capacity and the variety of backgrounds that chameleons must match in order to be camouflaged. Furthermore, species that showed only limited color change live in open habitats with dense ground vegetation such as grasslands and heaths. But this might not be simply due to predation, because the display colors of these species are just as conspicuous to bird predators as the display colors of species in other habitats. Together, these results support the hypothesis that dramatic color change evolved in dwarf chameleons largely as a strategy to facilitate social signaling rather than camouflage.

The authors suggest that limited color change might have evolved originally to function in thermoregulation or camouflage but that the dramatic changes in hue shown by some chameleon species evolved subsequently to enable chameleons to communicate using bright, flashy colors. These findings have broader implications for the evolution of animal social signals, since they support the importance of selection for signals that are highly detectable within the animal's environment and demonstrate how studies that take animal visual systems into account can be used to understand the evolution of signal diversity in animals.
